# Internal limiting membrane separation and posterior vitreous hyperreflective dots: novel OCT findings in Purtscher-like retinopathy

**DOI:** 10.1186/s12886-024-03413-w

**Published:** 2024-03-27

**Authors:** Vishma Prabhu, Aishwarya Joshi, Sai Prashanti Chitturi, Naresh Kumar Yadav, Jay Chhablani, Ramesh Venkatesh

**Affiliations:** 1 Department of Retina and Vitreous, Narayana Nethralaya, #121/C, 1st R Block, Chord Road, Rajaji Nagar, 560010 Bengaluru, Karnataka India; 2 Medical Retina and Vitreoretinal Surgery, University of Pittsburgh School of Medicine, 203 Lothrop Street, Suite 800, 15213 Pittsburg, PA USA

**Keywords:** Purtscher retinopathy, Inflammation, Steroids, Internal limiting membrane separation, Posterior vitreous hyperreflective dots

## Abstract

**Background:**

Purtscher or Purtscher-like retinopathy is diagnosed by retinal hemorrhages and areas of retinal whitening on fundus examination, as well as a reduction in visual acuity due to microvascular occlusion of the precapillary retinal arterioles. We describe novel optical coherence tomography (OCT) findings of internal limiting membrane (ILM) separation and posterior hyperreflective dots in a case of Purtscher-like retinopathy in this report.

**Methods:**

A 33-year-old man with acute pancreatitis and alcohol-induced liver disease presented to the retina department complaining of four days of painless vision loss in both eyes. Both eyes’ anterior segment examination and intraocular pressure were normal. Dilated fundus examination of both eyes revealed confluent areas of retinal whitening, hemorrhages, and cotton-wool spots over the posterior pole, indicating Purtscher-like retinopathy. OCT scans through the macula revealed dense inner retinal reflectivity, thickening, and loss of retinal layer stratification, as well as outer retinal layer shadowing and islands of ILM separation, posterior vitreous hyperreflective dots, and minimal subfoveal fluid, all of which corresponded to areas of retinal whitening on fundus photographs. The patient was given a brief course of systemic steroids.

**Results:**

On the tenth day after the presentation, visual acuity in the right eye had improved to 6/18 and finger counting at 1 m in the left eye. The retinal findings had faded. The retina had reverted to its normal thickness on the OCT scans, with minimal hyperreflectivity remaining. The ILM separation and posterior vitreous hyperreflective dots were no longer present.

**Conclusion:**

Following Purtscher or Purtscher-like retinopathy, we believe inflammation could play a major role in the development of these two novel OCT findings. This case offers an additional perspective on the underlying mechanisms responsible for the retinal manifestations observed in Purtscher or Purtscher-like retinopathy.

## Introduction

The initial description of Purtscher’s retinopathy was documented by Otmar Purstcher in 1910 in a patient who had experienced a head injury resulting from a fall from a tree. The fundus exhibited retinal hemorrhages and regions of retinal whitening, with reduction in visual acuity [1]. Purtscher-like retinopathy is a term used to describe the observation of comparable retinal manifestations in cases unrelated to trauma, most frequently associated with acute pancreatitis, renal failure, disseminated intravascular coagulopathy, thrombotic thrombocytopenic purpura, and autoimmune diseases [2]. The pathogenesis typically centers on the occlusion of the precapillary retinal arterioles at the microvascular level [3]. However, it is worth noting that choroidal vascular abnormalities have also been documented in the existing literature [4, 5]. The acute stage of the disease in optical coherence tomography (OCT) exhibits distinct features, including increased reflectivity in the inner retinal layers, which is accompanied by shadowing and obscuring of the outer retinal layers [6]. Additionally, there may be varying levels of macular edema present. During the chronic phase of the disease, there exists a varying extent of damage to the outer retinal layer and subsequent loss of the photoreceptor layer, which contributes to the observed reduction in vision in affected individuals [7]. The presence of internal limiting membrane (ILM) separation and posterior vitreous hyperreflective dots in cases of acute Purtscher’s retinopathy has not been documented in the existing literature. However, these findings have been observed in individuals experiencing acute central retinal artery occlusion [8, 9]. 

This case report presents novel observation of ILM separation and posterior hyperreflective dots using OCT in a classic case of Purtscher-like retinopathy subsequent to acute pancreatitis and alcohol-induced liver disease. The report also documents the temporal evolution of these findings.

## Case presentation

**Fig. 1:**
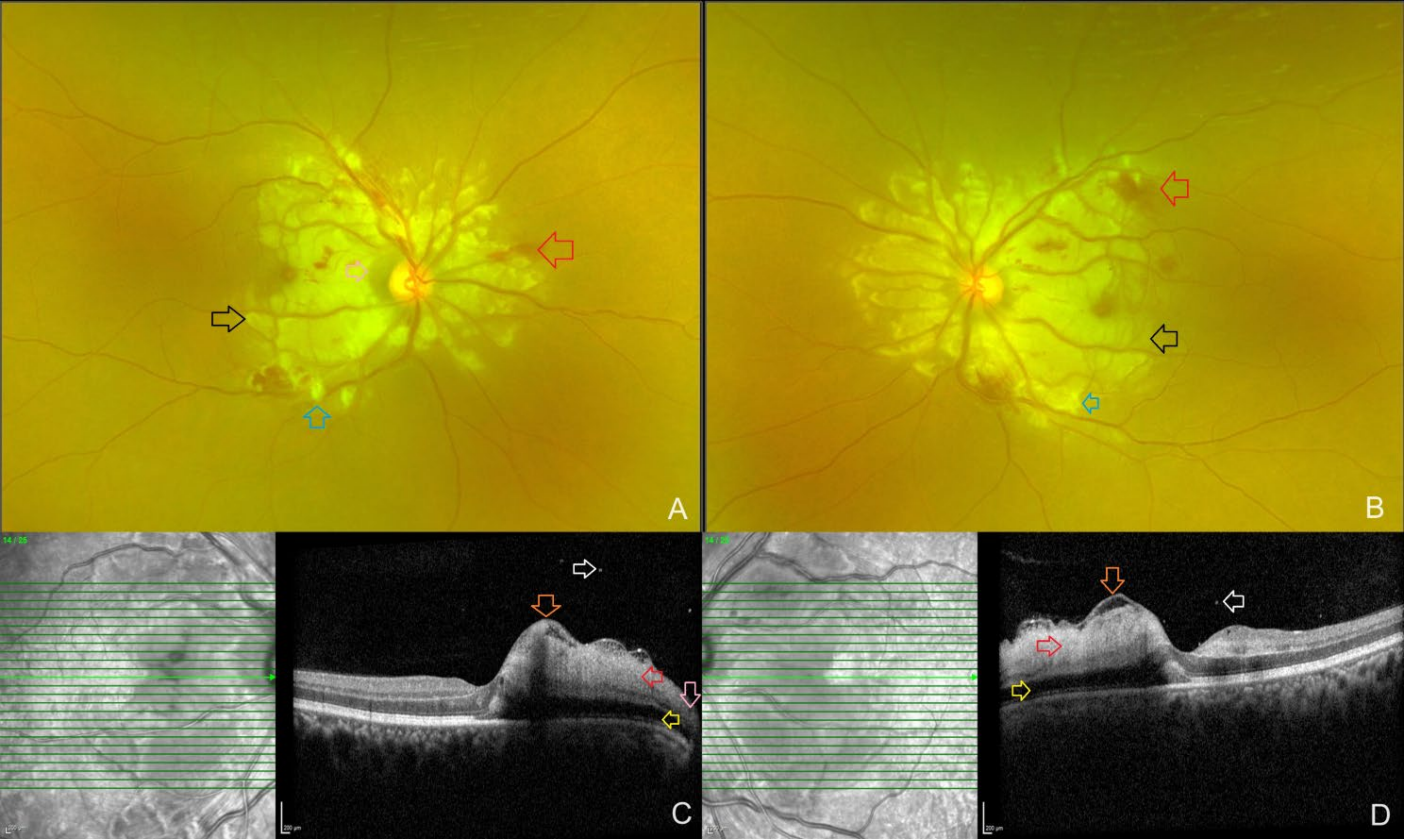
Clinical and optical coherence tomography (OCT) findings of a 33-year-old man diagnosed with Purtscher-like retinopathy at presentation: **A**, **B**: Dilated fundus examination of both eyes shows confluent polygonal areas of retinal whitening (black arrow) and a small number of retinal hemorrhages (red arrow) and some cotton-wool spots (blue arrow) over the posterior pole and on both sides of the optic disc. The remainder of the retinal periphery and the optic nerve head appeared normal. Sparing of the retina at the papillomacular bundle was noted (pink arrow). **C**, **D**: Spectralis (Heidelberg Engineering, Germany) OCT scans were performed using the horizontal line-raster protocol for both eyes over the macula. The horizontal line scans passing through the macula revealed dense inner retinal opacification and thickening and loss of inner retinal layer stratification (red arrow), as well as outer retinal layer shadowing (yellow arrow). In addition, areas of retinal opacification in both eyes exhibited islands of ILM separation (orange arrow) and posterior vitreous hyperreflective dots (white arrow) and minimal subfoveal fluid. Sparing of the retinal layers at the papillomacular bundle adjacent to the temporal margin of the optic disc was noted in the right eye (pink arrow)

**Fig. 2:**
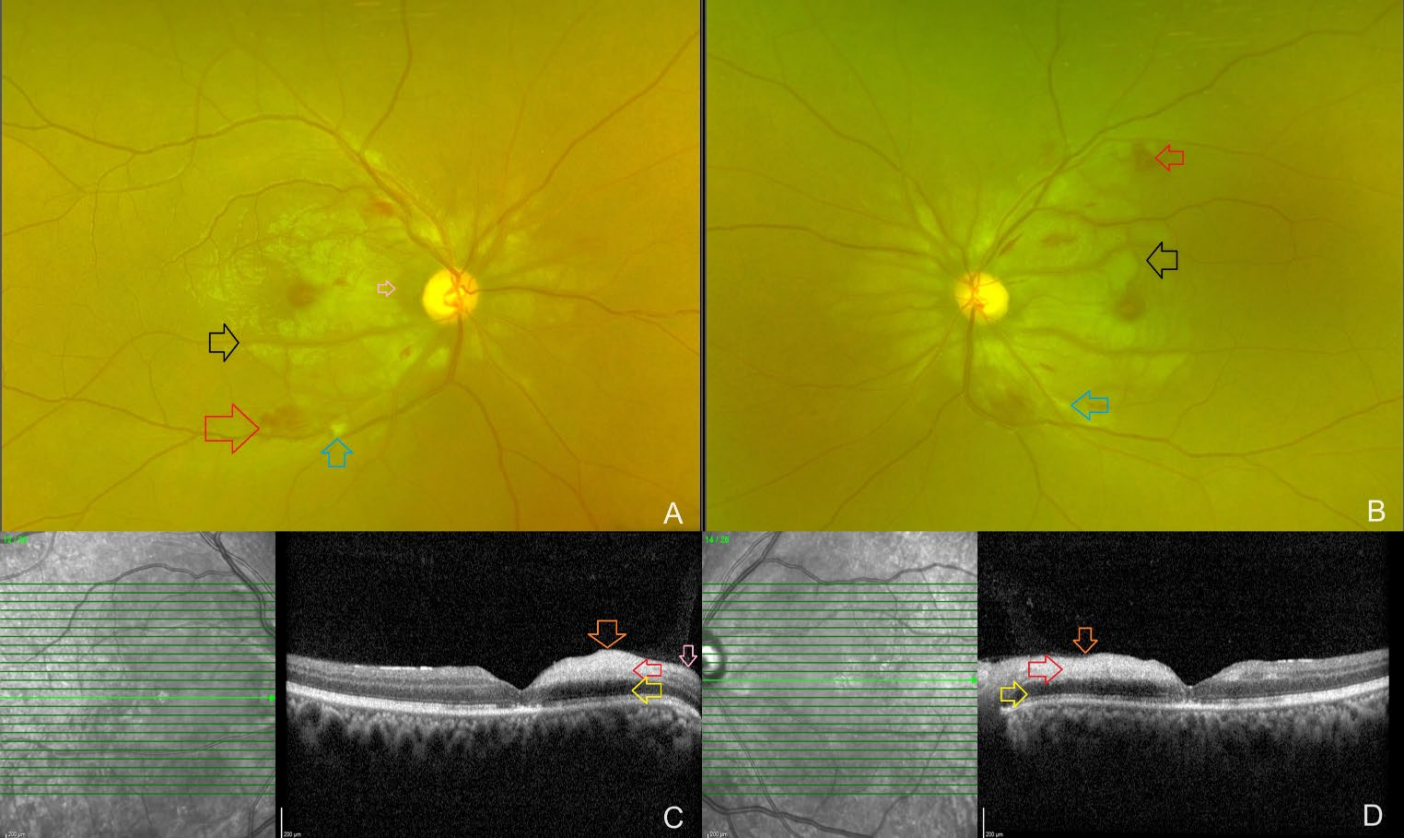
Clinical and optical coherence tomography (OCT) findings, ten days after presentation: **A**, **B**: Clinical fundus photographs shows fading of the retinal whitening areas (black arrow), few retinal hemorrhages (red arrow) and minimal cotton-wool spots (blue arrow). Sparing of the retina at the papillomacular bundle was noted in the right eye (pink arrow). **C**, **D**: On the OCT scans, the retinal thickness had reduced with minimal retinal opacification present. The inner retinal layer stratification was beginning to get restored (red arrow). The retinal layers at the papillomacular bundle adjacent to the temporal edge of the optic disc was unaffected in the right eye (pink arrow). The outer retinal layer’s shadowing had also diminished (yellow arrow). The separation of the ILM observed during the initial visit had disappeared (orange arrow)

A 33-year-old male presented to the retina department of an eye hospital with complaints of sudden, painless vision loss in both eyes for the past four days. Three weeks ago, he was diagnosed with acute pancreatitis and alcohol-induced liver disease and was admitted to a local hospital for treatment. His right and left eye visual acuity was 6/120 and counting fingers close to the face, respectively. On systemic examination, his vital parameters were normal. The intraocular pressure and anterior segment examination of both of his eyes were within normal limits upon ocular examination. Examination of the dilated fundus of both eyes revealed confluent areas of retinal whitening and a small number of retinal hemorrhages and few cotton-wool spots over the posterior pole and on both sides of the optic disc (Fig. 1A, B). The remainder of the retinal periphery and the optic nerve head appeared normal. The clinical findings were recorded using an Optos® (Daytona, U.S.A) ultrawide field fundus camera. Spectralis (Heidelberg Engineering, Germany) OCT scans were performed using the horizontal line-raster protocol for both eyes over the optic nerve head and macula. The horizontal line scans passing through the macula revealed dense inner retinal reflectivity and thickening and loss of inner retinal layer stratification, as well as outer retinal layer shadowing, which corresponded to the areas of retinal whitening on the fundus photographs. In addition, areas of retinal opacification in both eyes exhibited islands of ILM separation and posterior vitreous hyperreflective dots and minimal subfoveal fluid. An interesting finding on the horizontal macular OCT scan of the right eye was the sparing of a small portion of the papillomacular bundle from retinal opacification, whereas in the left eye, retinal opacification extended to the temporal margin of the optic disc and involved the papillomacular bundle (Fig. 1C, D). The patient was diagnosed with Purtscher’s-like retinopathy due to acute pancreatitis and alcohol-induced liver disease based on the available clinical history, clinical, and retinal imaging findings. After receiving clearance from our in-house physician, a course of systemic steroids was planned for the patient due to his poor vision in both eyes and possible optic nerve dysfunction due to reduced perfusion to the optic nerve. The visual evoked potential test was not used in this case. The physician performed blood tests that revealed normal serum amylase [133 U/L; Range: 40–140 U/L] and elevated alanine transaminase [49 U/L; Range: 4-36U/L], aspartate transferase [51 U/L; Range: 8-33U/L], and alkaline phosphatase [78 IU/L; Range: 44–147 IU/L] enzyme levels. The remaining liver function tests, including direct and indirect bilirubin levels, random blood sugar level, and a complete blood hemogram, were within normal limits. On the advice of the treating physician, the patient got started on a short course of systemic steroids (50 mg of Tab Wysolone daily, tapered by 10 mg every 5 days). Ten days after the patient’s initial presentation, his visual acuity had improved to 6/18 in the right eye and finger counting at 1 m in the left eye. The patient’s fundus photograph and OCT scans were repeated. This time, the whitening had begun to fade and there were only a few persistent retinal hemorrhages and cotton-wool spots (Fig. 2A, B). On the OCT scans, the retina had returned to its normal thickness while minimal hyperreflectivity remained. The inner retinal layer stratification was beginning to be restored. The outer retinal layer’s shadowing had also diminished. The separation of the ILM and the posterior hyperreflective vitreous dots observed during the initial visit had disappeared (Fig. 2C, D). Tapering dose of systemic steroids was continued, and a 3-week follow-up appointment was scheduled. The patient’s informed consent was obtained for the publication of his clinical information and retinal images.

## Discussion

The current view regarding the retinal findings in Purtscher or Purtscher-like retinopathy is centered on the microembolization of the precapillary arterioles, which affects the retinal microvasculature [3, 10]. The occurrence of embolic occlusion can be attributed to various factors, such as the presence of fat emboli resulting from long bone fractures, the dissemination of pancreatic proteases in the systemic circulation observed in cases of acute pancreatitis, and the activation of C5 and complement factors associated with renal failure and other autoimmune diseases, which ultimately leads to leucocyte aggregation [11–13]. In cases where the emboli are of significant size, they have the potential to obstruct the proximal segment of retinal arterioles, leading to the extensive whitening of the retina observed in individuals diagnosed with branch retinal artery occlusion. Conversely, when the emboli are smaller in size, they have the ability to obstruct the distal segment of retinal arterioles, resulting in the characteristic appearance of cotton-wool spots on fundoscopic examination. In the present instance, substantial contiguous regions of retinal whitening were observed in both eyes, indicating the presence of large emboli. Furthermore, we observed notable opacification of the inner retina, thickening of the inner retina, and disruption of the stratification of the inner retina layer, along with separation of the ILM and the presence of hyperreflective dots in the posterior vitreous in both eyes, as observed using OCT. These findings corresponded to the regions of retinal whitening observed during clinical examination. In instances of central retinal artery occlusion, comparable OCT observations have been documented, suggesting a higher degree of vascular occlusion [14]. Therefore, it can be inferred that the occlusion severity observed in our case of Purtscher-like retinopathy was substantial. The occurrence of ILM separation or detachment, as well as the presence of posterior vitreous hyperreflective dots, has been previously recorded in cases of more severe central retinal artery occlusion [8]. The authors put forth a pathogenesis model that suggests that inflammation caused by reperfusion injury is responsible for cellular damage in the inner retina. This damage then could lead to the separation of the ILM and the formation of hyperreflective dots in the posterior vitreous. The identical pathogenesis can be extrapolated to the present instance of Purtscher-like retinopathy. In cases of Purtscher retinopathy, high-dose corticosteroids, administered orally or intravenously, are commonly prescribed and have demonstrated favourable results [15]. Steroids possess the capacity to stabilize the neuronal membrane and microvascular channels that have been compromised, thereby facilitating a partial restoration of neuronal fibres that have not undergone irreversible damage. Steroids additionally impede the process of granulocyte aggregation and complement activation, both of which are pivotal in the development of this particular disease [16]. After administering oral systemic steroids, we observed the reattachment of the ILM separation and disappearance of posterior vitreous hyperreflective dots. This supports a possible hypothesis that inflammation resulting from microvascular occlusion could have been the likely underlying cause for the novel OCT findings of ILM separation and posterior vitreous hyperreflective dots in this particular case. The observed discrepancy in visual acuity improvement between the two eyes in our study may be primarily attributed to the varying levels of occlusion severity in each eye and the preservation of papillomacular bundle perfusion in the right eye. Additionally, this difference could potentially be influenced by the varying extent of damage to the outer retinal layer in both eyes. However, additional retinal imaging with fluorescein angiography or optical coherence tomography angiography could have helped to determine the extent of retinal ischemia and a possible explanation for the disparities in visual acuity changes in both eyes.

In summary, this case offers an additional perspective on the underlying mechanisms responsible for the retinal manifestations observed in Purtscher or Purtscher-like retinopathy. The involvement of inflammation in the retinal manifestations of this disease is of significant importance, and the administration of steroids may offer potential benefits in terms of early restoration of retinal structure and improvement in visual acuity.

## Data Availability

The datasets used and/or analysed during the current study are available from the corresponding author on reasonable request.

## Abbreviations

OCT: optical coherence tomography
ILM: internal limiting membrane
